# Double Lung Transplantation for Idiopathic Pulmonary Fibrosis in a Patient with a History of Liver Transplantation and Prolonged Journey for Disease-Specific Antifibrotic Therapy

**DOI:** 10.1155/2022/4054339

**Published:** 2022-08-13

**Authors:** Sebastian Majewski, Maria Królikowska, Ulrich Costabel, Wojciech J. Piotrowski, Marek Ochman

**Affiliations:** ^1^Department of Pneumology, Medical University of Lodz, 90-153 Lodz, Poland; ^2^Silesian Centre for Heart Diseases in Zabrze, Department of Cardiac, Vascular and Endovascular Surgery and Transplantology, Medical University of Silesia, 40-055 Katowice, Poland; ^3^Department of Pneumology, Ruhrlandklinik, Medical Faculty, University of Duisburg-Essen, 45239 Essen, Germany

## Abstract

Idiopathic pulmonary fibrosis (IPF) is characterized by uncontrolled progressive lung fibrosis with a median survival of 3 to 5 years. Although currently available pharmacotherapy cannot cure the disease, antifibrotics including pirfenidone and nintedanib were shown to slow disease progression and improve survival in IPF. Nevertheless, there is a knowledge gap on the safety of antifibrotics in patients after liver transplantation receiving concomitant immunosuppressive therapy. This case report of a 68-year-old male patient with IPF illustrates how a complex medical history has led to diagnostic and therapeutic challenges considerably affecting clinical decisions and impacting the patient's journey. The increasing severity of lung function impairment due to the progressive natural history of IPF ultimately led to severe respiratory failure. Double lung transplantation (LTx) was performed as the only therapeutic option in end-stage disease with the potential to improve quality of life and survival. To the best of our knowledge, this is the first case report describing the feasibility and safety of antifibrotic therapy with pirfenidone for IPF in a 68-year-old patient with a history of liver transplantation receiving concomitant immunosuppressive therapy with tacrolimus who underwent successful double lung transplantation when alternative medical interventions had been exhausted.

## 1. Introduction

Idiopathic pulmonary fibrosis (IPF) is a chronic, progressive, fibrotic lung disease of unknown etiology characterized by the radiographic and histopathologic pattern of usual interstitial pneumonia (UIP). It is known to have outcomes worse than many cancers, with a median survival of 3–5 years after diagnosis, although the disease course varies significantly in individuals [1]. Repetitive and chronic injuries leading to subsequent dysfunction of the alveolar epithelium are central for the initiation of the pathogenic process in IPF. The exact trigger of IPF is unknown; nevertheless, a few risk factors including genetic predisposition, environmental pollutants, microaspirations, cigarette smoking, certain viral infections, and occupational exposures (wood and metal dust) are considered associated with IPF [2–8]. The reported IPF prevalence ranges from 0.5 to 27.9/100.000 and the incidence from 0.22 to 8.8/100.000 [9]. Moreover, IPF incidence is increasing worldwide [10]. Prevalence and incidence are higher in men than in women and increase with age, the disease typically occurring over the age of 50 [1, 11]. The UIP pattern, observed on high-resolution computed tomography (HRCT) and histopathology, is characteristic for IPF but not pathognomonic. The diagnosis of IPF is based on typical HRCT findings of a UIP pattern accompanied by a compatible clinical context and after careful exclusion of other known causes for lung fibrosis. In patients suspected of IPF who present with other than definite UIP patterns on HRCT, additional diagnostic procedures including surgical lung biopsy (SLB) are recommended [12, 13]. Although no curative drug therapy for IPF has been found, two antifibrotics, pirfenidone and nintedanib, proved to slow the disease progression and to have a mortality benefit [14, 15]. However, antifibrotic therapy may be associated with many adverse drug reactions (ADR) and tolerability issues resulting in considerable treatment discontinuations. Besides disease-modifying drugs, multifaceted IPF management embraces patient education, psychosocial support, smoking cessation, management of comorbidities, supplemental oxygen therapy, pulmonary rehabilitation, symptom management and palliative care, and interventions taken in acute exacerbation of IPF (AE-IPF) [16]. In patients with advanced end-stage respiratory failure, when the above medical interventions have been exhausted, the only management option with the potential to improve quality of life and survival is lung transplantation [17].

## 2. Case Presentation

This is a case report on a 68-year-old man who underwent double lung transplantation in February 2020 due to advanced respiratory failure in the course of IPF. The patient's medical history relevant to lung fibrotic disease dates to 2013 when during the diagnostic evaluation before the planned liver transplantation, lung fibrosis was detected for the first time on chest CT while the patient was still asymptomatic. Previous exposures relevant for this case included a 15 pack-year history of smoking and a distant three-year period of parrot breeding. His past medical history included type 2 diabetes, gastroesophageal hernia, paroxysmal atrial fibrillation, and several endoscopic sclerosing interventions for esophageal varices. In 2014, the patient underwent a liver transplantation procedure for advanced toxic hepatocirrhosis. After the successful liver transplant, the patient was maintained on immunosuppressive therapy with tacrolimus. In 2017, the patient started to complain of major effort dyspnea and cough. Physical examination at that time revealed bilateral crackles at the base of the lungs on auscultation and finger clubbing. The HRCT showed extensive bilateral interstitial fibrosis with peripheral basilar predominant reticular opacities accompanied by peripheral traction bronchiectasis and subpleural honeycombing. The findings were compatible with the criteria of a definite UIP pattern. It is of note that the patient did not have any family history of lung fibrosis. Detailed differential diagnosis towards autoimmune diseases and hypersensitivity pneumonitis (HP) was conducted showing negative serological results. At this point of time, the exclusion of known causes of interstitial lung fibrosis with concurrent clinical and radiological context prompted the suspicion of IPF. Nevertheless, IPF was not confirmed until 2019, when a multidisciplinary team at one of the Polish reference centers for interstitial lung diseases (ILD) ultimately made a confident diagnosis of IPF. Despite the available antifibrotic therapy in Poland reimbursed by the National Health Fund, the treatment was not initiated due to the lack of data on the safety of antifibrotic drugs in patients who had undergone liver transplantation and were receiving concomitant treatment with tacrolimus; as such patients were not participating in the pivotal clinical trials of the two antifibrotics. It is of note that both drugs, pirfenidone and nintedanib, may have potential hepatotoxic adverse effects, and data on possible interactions with tacrolimus are also lacking.

The next step in the prolonged patient journey was the second opinion at one of the European university centers for ILD in March 2019. Conclusions from the second opinion consultation confirmed the correctness of the patient's IPF diagnosis and lack of data on the safety of antifibrotic treatment in patients after liver transplantation and receiving concomitant immunosuppressive therapy with tacrolimus. The contact with both antifibrotic drug manufacturers confirmed a knowledge gap in this clinical situation. Nevertheless, given the poor prognosis in IPF and the lack of alternative treatment options, a trial of antifibrotic therapy with a more vigilant approach was proposed. Following the second opinion consultation, the patient contacted another Polish university reference center for ILD, where after careful case evaluation, he was enrolled in the antifibrotic treatment program with pirfenidone in May 2019. At this point, his forced vital capacity (FVC) was 76% of the predicted value (% pred.) and the single-breath transfer factor of the lung for carbon monoxide (TL,_CO_) was 38% pred. Resting oxygen saturation (SpO_2_) was 97%, but he desaturated to SpO_2_ 76% in the six-minute walk test (6MWT) after 390 meters covered with 3 points on the Borg dyspnea scale at the end of the test. Taking into consideration the function of the transplanted liver and concomitant therapy with tacrolimus, the initial low dose of 267 mg pirfenidone 3 times daily was initiated under weekly monitoring of the serum liver parameters and of tacrolimus whole blood levels. For safety reasons, the decision for a longer than recommended drug titration period was made. With no relevant deterioration of liver function parameters and good drug tolerance, the dose was escalated to 534 mg 3 times daily after 4 weeks. The full recommended dose of 801 mg of pirfenidone given 3 times daily was reached after 8 weeks, without any safety signals or drug intolerance symptoms. Exertional desaturations below 90% were treated with a portable source of supplemental oxygen.

Despite the initiated antifibrotic therapy, the patient's general condition was slowly deteriorating. By the lack of other therapeutic options, a decision of the patient's preliminary qualification for lung transplantation was made. The patient was referred to the lung transplantation center in June 2019 for specialist consultation. Upon assessment at the lung transplantation center, pulmonary function tests revealed restrictive lung disorder with the following results: FVC of 72% pred., total lung capacity (TLC) of 68% pred., residual volume (RV) of 56% pred., RV/TLC ratio of 81%, and TL,_CO_ of 32% pred. During the 6MWT, the patient reached the distance of 420 m with 4 points on the Borg scale and 14% desaturation (from 93% to 79%) during the test. Echocardiography indicated the probability of pulmonary hypertension (right ventricle systolic pressure of 60 mmHg, acceleration time of 72 ms, tricuspid annular plane systolic excursion of 22 mm, left ventricle ejection fraction of 59%), which was confirmed in a consecutive right heart catheterization procedure by the mean pulmonary artery pressure of 29 mmHg. Ultimately, the patient was considered preliminarily qualified for double lung transplantation and complementation of further procedure-related investigations was recommended.

After six months since the initiation of pirfenidone therapy, the patient decided on his own to discontinue the medication due to a progressive decrease of appetite, noted from the third month of treatment, and loss of weight. Four weeks later, he was admitted to the local hospital due to acute respiratory deterioration with increased dyspnea, tachypnea, productive cough, and resting SpO_2_ of 55%. The patient's serious condition demanded endotracheal intubation and mechanical ventilation. Chest radiography revealed bilateral, irregular interstitial opacifications. Based on the clinical suspicion of community-acquired pneumonia (CAP), empirical antibiotic therapy with ceftriaxone was implemented together with methylprednisolone intravenously. Over five consecutive days, the patient's condition improved, and he was discharged home with respiratory failure at rest demanding long-term home oxygen therapy. Two months after the acute episode of respiratory hospitalization with significant deterioration of lung function, the patient was admitted to the transplantation unit of the Silesian Center for Heart Diseases due to the availability of a compatible lung donor. Uncomplicated sequential double lung transplantation (LTx) was performed on February 14, 2020, followed by immunosuppressive maintenance therapy including tacrolimus, mycophenolate mofetil, and prednisone. Additionally, prophylaxis of cytomegalovirus and fungal infections with valganciclovir and voriconazole was introduced. Six weeks after the surgery, the patient was successfully discharged from the hospital in a good general condition. The patient's pulmonary function had significantly improved, as shown in the spirometry four months after LTx with the forced expiratory volume in 1 second (FEV_1_) of 97% pred. and FVC of 84% pred. During the 6MWT, the patient covered the distance of 265 m with 1 point on the Borg scale and without desaturation throughout the test. Currently, 2 years after LTx, the patient remains in good general condition without any posttransplant medical complications.

## 3. Discussion

Our case report illustrates real-life diagnostic and management challenges and confirms considerable diagnostic and treatment delays in IPF [18, 19]. The complex past medical history of the patient, including a short period of parrot breeding and a liver transplantation procedure with concomitant immunosuppressive treatment, considerably affected clinical decisions and prolonged the patient's journey to disease-specific antifibrotic therapy of IPF. Finally, adverse drug reactions related to pirfenidone treatment resulted in treatment discontinuation. The progressive natural history of the disease with worsening of pulmonary function after exhaustion of other medical interventions prompted successful double lung transplantation which led to an increase of quality of life and survival benefits.

Although IPF is a rare disease, physicians should consider IPF as a potential cause of unexplained exertional dyspnea and/or cough in elderly patients and refer them to a pulmonologist for further evaluation. Establishing an accurate diagnosis of IPF requires specialist expertise in ILD diagnosis [12, 13]. In our case, the delay of a correct diagnosis of IPF from the first incidental finding of asymptomatic lung fibrosis documented on the chest CT performed before liver transplantation to the confirmation of IPF at the reference ILD center was close to 6 years. Taking into account only the time from the first disease symptoms to the confirmed diagnosis of IPF, the delay was close to 2 years. In a published survey undertaken among European IPF patients, the median reported time from initial presentation to confirmed diagnosis of IPF was 1.5 years (range <1 week to 12 years) and in 58% of cases, a delay of >1 year between initial presentation and a confirmed diagnosis of IPF was reported [19]. In a recent observational study on the Polish experience with pirfenidone therapy in a large cohort of patients with IPF (the PolExPIR study), the median time from the first symptoms to IPF diagnosis was 15.5 months [20]. Both national Polish and European findings confirm the urgent need for improvement in the area of ILD diagnosis. Multidisciplinary discussion (MDD), involving at minimum a pulmonologist and a radiologist with expertise in the differential diagnosis of ILD, is required to ensure an accurate diagnosis [12, 21]. MDD is critical for the diagnosis of IPF and other ILD, especially when the HRCT patterns and clinical workup do not provide a clear diagnosis [22]. In our case, the first ILD workup performed at the local respiratory center was a reason for an initial delay of an accurate IPF diagnosis. A recent survey undertaken in a representative group of Polish pulmonologists showed that only 63% of them routinely work with an ILD expert radiologist for the differential diagnosis of ILD [21]. In the authors' opinion, the formation of experienced multidisciplinary teams in Polish respiratory centers involved in ILD diagnosis and management should become a priority as long as IPF diagnostic standards are considered. Prompt diagnosis of IPF is important to enable patients to receive appropriate care already at an early stage, which translates into the best patients' outcomes [23].

Our case was even more challenging due to the history of liver transplantation and concomitant immunosuppressive therapy with tacrolimus. Despite confirmation of the IPF diagnosis at the reference ILD center, the decision regarding the initiation of antifibrotic therapy was postponed due to concerns of safety in such a clinical case scenario, since there were no data available on the safety of either pirfenidone or nintedanib in patients with IPF after liver transplantation and concomitant therapy with tacrolimus. This situation resulted in an additional delay in accessing treatment with the disease-modifying drug in our patient. A subsequent second opinion of our complex case at a European ILD center confirmed the accuracy of IPF diagnosis and the gap in the safety data of antifibrotic therapy in patients after liver transplantation and concomitant immunosuppression. Nevertheless, given the poor prognosis and the lack of alternative options, a pragmatic approach of a trial of antifibrotic therapy was proposed. Our case report confirms the feasibility and safety of pirfenidone therapy in such a case scenario. For safety reasons, the drug titration period was extended from 2 weeks to 8 weeks. Good drug tolerance and no safety signals were observed during that time. Despite the initial good tolerance of pirfenidone therapy, the patient decided to discontinue drug intake after 6 months because of the progressive decrease of appetite and loss of weight; both are known pirfenidone-related adverse events. In the recent aforementioned real-world data (RWD) study on the Polish experience of pirfenidone therapy in IPF, decreased appetite and weight loss were reported by around one-third of treated patients and the gastrointestinal ADR category was the most frequent reason for pirfenidone discontinuation [20]. Moreover, we cannot exclude that pirfenidone discontinuation was associated with the subsequent respiratory deterioration with the need for hospitalization and mechanical ventilation of our patient. It is of note that in a pooled analysis of phase 3 IPF randomized clinical trials (RCTs), patients receiving pirfenidone had a lower risk of nonelective respiratory-related hospitalizations over 1 year [24]. The high rate of treatment discontinuations observed in both RCTs and RWD studies of antifibrotics underscores the importance of supporting and guiding patients in the management of side effects so that they can adhere to their medications to ensure an optimal treatment benefit [14, 15, 18, 20, 25]. Dose adjustment (reduction or interruption and reuptake with a lower dose) is strongly recommended by experts for the management of side effects and not the immediate permanent drug discontinuation. In the PASSPORT study, more patients completed treatment following dose adjustment (38.8%) than those who had no dose adjustment (26.1%) [26].

Finally, the disease progression and respiratory deterioration prompted LTx. At this point when medical options have been exhausted, the only treatment left with the potential to improve quality of life and survival is LTx [17]. The poor prognosis and the potential for an accelerated decline of lung function in IPF mean that early referral is crucial. Worldwide changes in donor lung allocation, including the Lung Allocation Score (LAS) in the USA and Eurotransplant, have significantly increased the LTx rates for candidates with ILD. IPF is now the leading indication for LTx worldwide [17, 27]. In our case, the patient was referred to a transplantation center after the initiation of antifibrotic therapy. The quick evaluation and lack of absolute contraindications resulted in the qualification to be included in the lung transplant waiting list. At the moment of his inclusion in the waiting list, the only relative contraindication was age above 65 years (the actual age of the patient at that time was 68 years). Age alone should not exclude a patient from receiving a lung transplant. The accelerated clinical deterioration after respiratory hospitalization was a direct trigger for a prompt decision regarding LTx in our patient. Currently, 2 years after the LTx procedure, the patient is in a stable clinical condition with evident improved quality of life and survival benefits.

## 4. Conclusions

The present case report illustrates how a complex medical history led to diagnostic and therapeutic challenges which considerably affected clinical decisions and impacted the IPF patient's journey. Moreover, our patient's history underscores the importance of MDD in establishing early and accurate diagnosis of IPF and access to appropriate IPF management without delay. Finally, to the best of our knowledge, this is the first case report describing the feasibility and safety of antifibrotic therapy with pirfenidone for IPF in a patient with a history of liver transplantation and concomitant immunosuppressive therapy with tacrolimus who underwent successful LTx.

## Data Availability

Additional anonymized data may be available on request. Please contact the corresponding author.

## References

[1] Raghu G., Collard H. R., Egan J. J. (2011). An official ATS/ERS/JRS/ALAT statement: idiopathic pulmonary fibrosis: evidence-based guidelines for diagnosis and management. *American Journal of Respiratory and Critical Care Medicine*.

[2] Allen R. J., Guillen-Guio B., Oldham J. M. (2020). Genome-wide association study of susceptibility to idiopathic pulmonary fibrosis. *American Journal of Respiratory and Critical Care Medicine*.

[3] Hobbs B. D., Putman R. K., Araki T. (2019). Overlap of genetic risk between interstitial lung abnormalities and idiopathic pulmonary fibrosis. *American Journal of Respiratory and Critical Care Medicine*.

[4] Majewski S., Piotrowski W. J. (2021). Air pollution—an overlooked risk factor for idiopathic pulmonary fibrosis. *Journal of Clinical Medicine*.

[5] Baumgartner K. B., Samet J. M., Coultas D. B. (2000). Occupational and environmental risk factors for idiopathic pulmonary fibrosis: a multicenter case-control study. *American journal of epidemiology*.

[6] Bédard Méthot D., Leblanc É., Lacasse Y. (2019). Meta-analysis of gastroesophageal reflux disease and idiopathic pulmonary fibrosis. *Chest*.

[7] Pulkkinen V., Salmenkivi K., Kinnula V. L. (2012). A novel screening method detects herpesviral DNA in the idiopathic pulmonary fibrosis lung. *Annals of Medicine*.

[8] Trethewey S. P., Walters G. I. (2018). The role of occupational and environmental exposures in the pathogenesis of idiopathic pulmonary fibrosis: a narrative literature review. *Medicina*.

[9] Kaunisto J., Salomaa E.-R., Hodgson U., Kaarteenaho R., Myllärniemi M. (2013). Idiopathic pulmonary fibrosis - a systematic review on methodology for the collection of epidemiological data. *BMC Pulmonary Medicine*.

[10] Hutchinson J., Fogarty A., Hubbard R., McKeever T. (2015). Global incidence and mortality of idiopathic pulmonary fibrosis: a systematic review. *The European Respiratory Journal*.

[11] Lederer D. J., Martinez F. J. (2018). Idiopathic pulmonary fibrosis. *The New England Journal of Medicine*.

[12] Raghu G., Remy-Jardin M., Myers J. L. (2018). Diagnosis of idiopathic pulmonary fibrosis. An official ATS/ERS/JRS/ALAT clinical practice guideline. *American Journal of Respiratory and Critical Care Medicine*.

[13] Piotrowski W. J., Bestry I., Białas A. J. (2020). Guidelines of the Polish Respiratory Society for diagnosis and treatment of idiopathic pulmonary fibrosis. *Advances in Respiratory Medicine*.

[14] King T. E., Bradford W. Z., Castro-Bernardini S. (2014). A phase 3 trial of pirfenidone in patients with idiopathic pulmonary fibrosis. *The New England Journal of Medicine*.

[15] Richeldi L., du Bois R. M., Raghu G. (2014). Efficacy and safety of nintedanib in idiopathic pulmonary fibrosis. *The New England Journal of Medicine*.

[16] Moran-Mendoza O., Colman R., Kalluri M., Cabalteja C., Harle I. (2019). A comprehensive and practical approach to the management of idiopathic pulmonary fibrosis. *Expert Review of Respiratory Medicine*.

[17] George P. M., Patterson C. M., Reed A. K., Thillai M. (2019). Lung transplantation for idiopathic pulmonary fibrosis. *The Lancet Respiratory Medicine*.

[18] Sköld C. M., Arnheim-Dahlström L., Bartley K. (2019). Patient journey and treatment patterns in adults with IPF based on health care data in Sweden from 2001 to 2015. *Respiratory Medicine*.

[19] Schoenheit G., Becattelli I., Cohen A. H. (2011). Living with idiopathic pulmonary fibrosis. *Chronic Respiratory Disease*.

[20] Majewski S., Białas A. J., Buchczyk M. (2020). A multicentre retrospective observational study on Polish experience of pirfenidone therapy in patients with idiopathic pulmonary fibrosis: the PolExPIR study. *BMC Pulmonary Medicine*.

[21] Majewski S., Lewandowska K., Martusewicz-Boros M. M., Piotrowski W. J. (2019). Diagnostic and treatment standards in idiopathic pulmonary fibrosis in the era of antifibrotic drugs in Poland: a real-world practice survey. *Advances in Respiratory Medicine*.

[22] Furini F., Carnevale A., Casoni G. L. (2019). The role of the multidisciplinary evaluation of interstitial lung diseases: systematic literature review of the current evidence and future perspectives. *Frontiers in Medicine*.

[23] Kang J., Han M., Song J. W. (2020). Antifibrotic treatment improves clinical outcomes in patients with idiopathic pulmonary fibrosis: a propensity score matching analysis. *Scientific Reports*.

[24] Ley B., Swigris J., Day B. (2017). Pirfenidone reduces respiratory-related hospitalizations in idiopathic pulmonary fibrosis. *American Journal of Respiratory and Critical Care Medicine*.

[25] Costabel U., Bendstrup E., Cottin V. (2014). Pirfenidone in idiopathic pulmonary fibrosis: expert panel discussion on the management of drug-related adverse events. *Advances in Therapy*.

[26] Cottin V., Koschel D., Günther A. (2018). Long-term safety of pirfenidone: results of the prospective, observational PASSPORT study. *ERJ Open Research*.

[27] Weill D. (2018). Lung transplantation: indications and contraindications. *Journal of Thoracic Disease*.

